# Clinicodermoscopic and immunohistochemical observation of hepatitis B virus-associated acquired bilateral telangiectatic macules in a Chinese man^☆^^☆☆^

**DOI:** 10.1016/j.abd.2020.08.027

**Published:** 2021-09-27

**Authors:** Zi-Wei Zhang, Hao Wu, Ke-Feng Tang, Yi-Ming Fan

**Affiliations:** Department of Dermatology, Affiliated Hospital of Guangdong Medical University, Zhanjiang, Guangdong, China

Dear Editor,

Acquired Bilateral Telangiectatic Macules (ABTM) was first reported as telangiectatic pigmented macules primarily on the upper arms in Korean patients with underlying Chronic Liver Disorder (CLD).^1^, ^2^, ^3^ To date 65 Korean cases and one Chinese case have been reported.^1^, ^2^, ^3^, ^4^ Dermoscopy was useful to improve its clinical diagnosis.^1^ We describe the clinicodermoscopic and immunohistochemical features of hepatitis B virus-associated ABTM in another Chinese patient.

A 48-year-old Chinese man presented with asymptomatic telangiectatic macules that commenced on the left arm 2.5 years ago and gradually extended to upper limbs and torso. He had a 30-year history of hepatitis B with mildly abnormal liver enzymes during the last three years. Liver enzymes reduced but telangiectasis remained unchangeable after a 1-month course of oral compound glycyrrhizin and glucurolactone. No further treatments were given thereafter. There was no history of smoking, drinking, diabetes, hypertension, and ataxia. On examination, multiple, irregular, dark red telangiectatic macules with negative Darier’s sign were mainly distributed on the chest and upper limbs (Fig. 1a) and less on the face and upper back. No spider nevus, palmar erythema, mucosal lesion, neither hepatosplenomegaly were noted. Dermoscopy revealed tortuous/arborizing vessels and diffuse brownish pigmentation (Fig. 1b). The leucocyte count was 10.2 × 10^9^/L with 90% neutrophils. Biochemical examination showed elevated alanine (86.5 U/L) and aspartate aminotransferases (69.9 U/L), and normal coagulation function, glucose, lipids, estradiol, and testosterone. HBsAg, HBeAg, and PreS1Ag were positive, but anti-nuclear antibody was negative.

**Figure 1:**
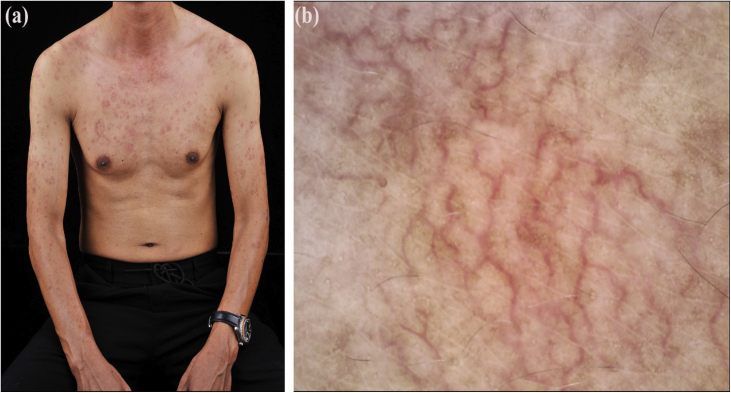
Clinicodermoscopic features of ABTM: (a), Telangiectatic erythematous macules on the chest and upper limbs. (b), Dermoscopy revealing tortuous/arborizing vessels and diffuse brownish pigmentation on the chest (×30).

Light Micrsoscopy displayed an unremarkable epidermis with basal hyperpigmentation, capillary proliferation, and dilated capillaries with mild infiltration of perivascular lymphohistiocytes in the upper dermis (Fig. 2). Giemsa stain and CD117 immunostaining demonstrated a few mast cells (Fig. 3a). Melan-A and S100 immunostaining found normal melanocytes in the epidermis (Fig. 3b). The patient was diagnosed with ABTM, and his lesions remained stable during a 16-month follow-up without specific treatment.

**Figure 2:**
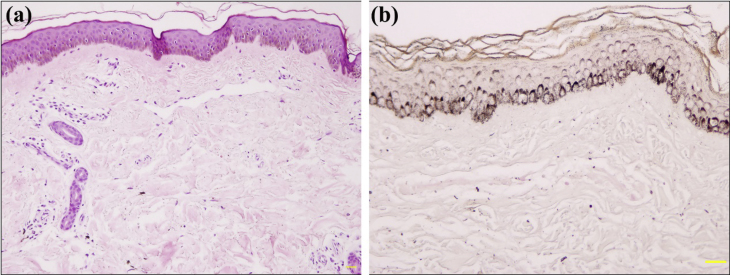
Histopathology of ABTM: (a), Basal hyperpigmentation, capillary proliferation, and dilated capillaries with mild infiltration of perivascular lymphohistiocytes in the upper dermis (Hematoxylin & eosin, ×100). (b), Hyperpigmentation in the basal layer of epidermis (Fontana-Masson, ×200).

**Figure 3:**
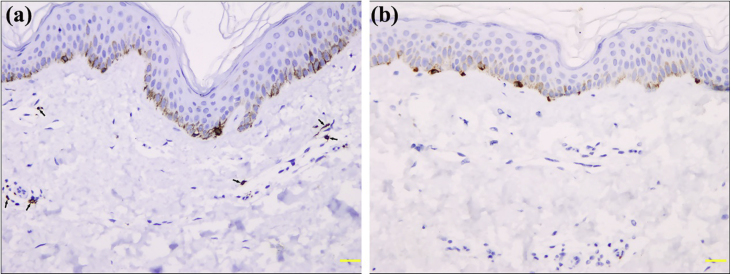
Immunohistochemistry of mastocytes and melanocytes in ABTM: (a), A few CD117 positive mast cells (arrows) around the dilated capillaries in the papillary dermis (×200). (b), Normal Melan-A positive melanocytes in the epidermis (×200).

ABTM, Acquired Bilateral Nevoid Telangiectasia (ABNT) and Telangiectasia Macularis Multiplex Acquisita (TMMA) are three newly described Acquired Bilateral Telangiectasia (ABT) in Korea and China. The causative factors include CLD, hypertension, diabetes, smoking, and so on, but true pathomechanism remains elusive. Both ABTM and ABNT are overwhelming in Koreans, while TMMA seems to be peculiar in Chinese. They frequently affect middle-aged men and are often associated with underlying diseases (especially CLD).^1^, ^2^, ^3^, ^4^, ^5^ Clinically, ABTM features two lesional components (brown pigmentation and telangiectasia) mostly on upper arms, ABNT manifests as superficial telangiectasia on the upper body, and TMMA presents as crops of telangiectatic vessels superimposed on erythematous macules on the arms and trunk.^1^, ^2^, ^3^, ^4^, ^5^ The widespread telangiectasia or trunk involvement are liable to have associated CLD.^1^, ^5^ Histologically, the key features of ABTM, ABNT, and TMMA are respectively dermal telangiectasias with epidermal hyperpigmentation; dermal telangiectasia with normal epidermis; and mild perivascular lymphocytic infiltration with or without telangiectasia.^1^, ^2^, ^3^, ^4^, ^5^ Absence of dermal telangiectasia might be due to vasoconstriction induced by epinephrine in local anesthetics.^5^ Collectively, the clinicopathological characteristics of these three ABT have not been well defined because of small samples, relatively heterogeneous patients, and clinical/morphological resemblance among the three diseases.^1^ Even if there are some histological differences: epidermal pigmentation, perivascular lymphocytic infiltrate, and dermal telangiectasia, they are minimal. They are hard to distinguish from each other in clinical practice because of their clinicopathological similarity or overlap. In line with the opinion of Kim et al., we believe that the three disorders are the same disease entity and propose the use of the Latin term “telangiectasia macularis multiplex acquisita”.^1^ In addition, Telangiectasia Macularis Eruptiva Perstans is similar to these entities, but histologically the presence of a mastocyte infiltrate can differentiate them.^1^

Dermoscopy displayed brown pigmentation, linear-irregular vessels, and angioid streak pattern in ABTM, corresponding to basal hyperpigmentation and dermal telangiectasia, respectively. Angioid streak pattern was defined as a central arteriole with superficial radiating small vessels, maybe representing a minor form of spider angioma.^1^ The severity and prevalence of angioid streak patterns were higher in ABTM patients with CLD than in those without CLD, but it was absent in our case.^1^

In conclusion, dermoscopy is useful to observe the inconspicuous pigmentation and telangiectasia in ABTM, but the potential value of the angioid streak pattern for the evaluation of underlying CLD remains to be verified. There is no convincing evidence to create several different names for ABT.

## Financial support

None declared.

## Authors' contributions

Zi-Wei Zhang: Conception and planning of the study; obtaining, analyzing, and interpreting the data; writing of the manuscript.

Hao Wu: Planning of the study; obtaining, analyzing, and interpreting the data; writing of the manuscript.

Ke-Feng Tang: Obtaining, analyzing, and interpreting the data.

Yi-Ming Fan: Conception and planning of the study, obtaining, analyzing, and interpreting the data, critical revision of the manuscript, and approval of its final version.

## Conflicts of interest

None declared.
